# Intra-shift affective dynamics in emergency unit workers: temporal deterioration and recovery windows

**DOI:** 10.3389/fpsyg.2026.1895858

**Published:** 2026-08-31

**Authors:** Rodolfo Mendoza-Llanos, Sebastián Maureira-Meneses

**Affiliations:** 1 Department of Social Science, School of Psychology, University of Bío-Bío, Chillán, Chile; 2 Adventist University of Chile, Chillán, Chile; 3 Ph.D. Program in Economics, Faculty of Business Sciences, University of Bío-Bío, Chillán, Chile

**Keywords:** affective dynamics, burnout, emotional dysregulation, healthcare workers, recovery strategies

## Abstract

Burnout research typically relies on global self-report measures that provide limited information about affective experience across the workday. Inspired by the Day Reconstruction Method, this exploratory study examined reconstructed intra-shift well-being–distress trajectories among 76 emergency healthcare workers. Participants completed a structured hourly reconstruction of a typical workday from T−2 to T14, together with measures of burnout and emotional dysregulation. Descriptive analyses, Pearson correlations, and sex-based mean comparisons were conducted, with false discovery rate corrections applied to the inferential analyses. The mean reconstructed trajectory showed an initial increase in well-being before and shortly after shift onset, a progressive decline across much of the shift, and an increase in affective valence during the post-shift period. All correlations between adjacent reconstructed time points remained significant after correction, providing exploratory support for short-range cross-time rank-order consistency. Although unadjusted correlations suggested less favorable ratings at selected time points among participants with higher burnout and emotional dysregulation scores, none remained significant after correction. Likewise, no time-specific sex comparison survived correction. These preliminary findings suggest that considering when affective declines occur across emergency shifts may help organizations identify candidate periods for brief support actions, such as supervisory check-ins, team support, or recovery opportunities. However, because the trajectories were retrospectively reconstructed, the timing and effectiveness of such micro-interventions should be evaluated in larger real-time studies before informing organizational practice.

## 1 Introduction

Historically, burnout research has used models focused on chronic stress, often relying on global questionnaires like the Maslach Burnout Inventory, Copenhagen Burnout Inventory, Oldenburg Burnout Inventory, or Burnout Assessment Tool. These instruments measure exhaustion, cynicism, and reduced efficacy, but not their variation during the workday (Edú-Valsania et al., 2022; Salvagioni et al., 2017; Nagle et al., 2024; Alanazy and Alruwaili, 2023). Typically used in two-point measurement designs, they estimate prospective health effects while treating burnout as stable, thereby obscuring changes in distress over the course of the day (Salvagioni et al., 2017; Edú-Valsania et al., 2022). In health research, recent reviews and studies report high burnout prevalence and links to stress, overload, trauma, and mobbing. However, they still rely on static measures that do not distinguish phases of the workday (Nagle et al., 2024; Izdebski et al., 2023; Cohen et al., 2023).

In parallel with this work, the literature on affective dynamics has shown that not only does the average level of affect matter, but also its variability, instability, and short-term inertia—that is, the degree to which emotional states are maintained or change between consecutive moments (Houben et al., 2015; Koval et al., 2013; Nelson et al., 2020). Building on these findings, a meta-analysis of intensive longitudinal studies showed that lower psychological well-being may be associated with specific affective patterns, including greater variability, instability, and persistence of negative affect (Houben et al., 2015). Importantly, although affective changes may reflect adaptive adjustment processes in response to situational demands, excessive affective instability or sustained negative affect may be associated with impaired affective recovery and poorer psychological functioning. Further supporting this perspective, studies using controlled experience and task sampling have found that depressive symptoms are linked to greater inertia and variability in negative affect, both in the laboratory and in daily life.

Together, these findings indicate that affective experience may show short-range continuity while also changing in average intensity or direction across longer periods (Koval et al., 2013; Nelson et al., 2020). These properties are not contradictory: temporal continuity refers to the extent to which affect at one moment is associated with affect at a nearby moment, whereas trajectory change refers to broader increases or decreases in affective intensity over time. Nevertheless, despite explicit calls to apply this temporal approach—focusing on intraindividual variability, reactivity, cycles, and inertia—to organizational outcomes, much of workplace research continues to treat these as attributes of the individual, without sufficiently specifying the particular work situations and moments that generate them (Diener et al., 2020; Brose et al., 2020).

Building on these considerations, occupational health studies using intensive methods such as ecological momentary assessment (EMA) or daily diaries have linked patterns of everyday emotion regulation (e.g., rumination, suppression, reappraisal) to affect trajectories over minutes, hours, or days. These findings show relationships between regulatory strategies and well-being. However, these studies rarely focus on intra-shift trajectories in high-demand work like emergency services, nor do they often include measures of burnout or emotional dysregulation to identify critical phases of affective decline. Methodological reviews of intraindividual affective variation highlight the lack of consensus regarding appropriate within-person measurement approaches and caution against using instruments designed primarily to assess between-person differences. This underscores the need for designs and measures capable of capturing changes in affective experience across specific periods of the workday (Brose et al., 2020).

To address some of these gaps, structured retrospective reconstruction methodologies for daily affective experience, inspired by the Day Reconstruction Method (DRM), have become relevant as a complementary alternative to real-time recordings (Kahneman et al., 2004). DRM was designed to combine the benefits of time-use studies and experience sampling. It allows participants to systematically reconstruct the previous day’s activities and experiences as episodes, using procedures to reduce recall bias (Kahneman et al., 2004; Anusic and Lucas, 2016). Comparative studies using Ecological Momentary Assessment (EMA) and the DRM suggest that both approaches can produce similar estimates of average affective intensity for experiences such as happiness, fatigue, and stress (Kim et al., 2013). They have also shown moderate-to-high between-person correlations and broadly comparable patterns of affective variation across different periods of the day (Dockray et al., 2010; Lucas et al., 2021). However, convergence appears to be stronger for average affect levels than for indicators of within-person variability and short-term affective dynamics.

In addition to the temporal organization of work demands, affective experience may vary according to time-of-day and circadian influences. Previous studies have identified differences in emotional experience between morning and evening periods (English and Carstensen, 2014), while experimental evidence suggests that circadian off-peak periods may be associated with greater emotional reactivity and less effective emotion regulation (Tucker et al., 2012). More broadly, circadian disruption has been linked to changes in mood and mental health, including in contexts involving shift work (Walker et al., 2020). These findings highlight the importance of distinguishing temporal patterns associated with the work shift from effects attributable to clock time or circadian processes.

Even so, combining retrospective and contextual recordings in real-world work settings is feasible and useful for identifying stressful events, stressor categories, and shift-to-shift differences. This enhances the ecological value of these methods for mapping affective and stress experiences across shifts (Doran et al., 2023). These approaches reduce participant burden and use structured episodic recall, making them especially relevant in high-demand work where real-time recording may be impractical or disruptive (Anusic and Lucas, 2016; Bakker et al., 2012; Diener and Tay, 2014). Structured time reconstruction further helps explore how well-being and illness are organized over time at work and links time profiles with burnout and emotional dysregulation, identifying candidate periods for future evaluation of prevention and intervention strategies (Schneider et al., 2020; Boemo et al., 2022; Doran et al., 2023).

Previous research indicates that burnout is associated with less favorable experiences of occupational well-being (Salvagioni et al., 2017; Izdebski et al., 2023). Research on affective dynamics further suggests that affective experience may exhibit short-range continuity between nearby moments while also showing broader changes in intensity or direction over time (Houben et al., 2015; Koval et al., 2013; Nelson et al., 2020). Examining these distinct temporal properties provides the conceptual basis for exploring reconstructed affective trajectories across the work shift. This evidence justifies the research approach. Thus, the overall objective of this study is to describe reconstructed hourly intra-shift trajectories of well-being and distress among emergency workers under intense demand. From an exploratory and hypothesis-generating perspective, the analyses were guided by the following exploratory hypotheses:

*H1*: Reconstructed well-being–distress ratings will show greater cross-time rank-order consistency between adjacent time points than between temporally distant time points.

*H2*: Higher burnout will be associated with less favorable reconstructed well-being–distress ratings at specific moments across the workday.

*H3*: Higher emotional dysregulation will be associated with less favorable reconstructed well-being–distress ratings across the workday, particularly during the early and later stages of the shift.

Given previous evidence of sex-related differences in burnout and psychological distress among emergency and healthcare workers (Diao et al., 2024; Purvanova and Muros, 2010), exploratory sex-based analyses of intra-shift trajectories were also conducted.

## 2 Methods

### 2.1 Design

A quantitative, descriptive, and correlational study was conducted. It was based on a structured retrospective reconstruction of daily affective experience over an hourly period. The design aimed to describe the intra-shift temporal organization of subjective well-being and distress in emergency workers. It explored patterns of affective continuity and associations with indicators of psychological well-being and occupational distress. The procedure is conceptually inspired by approaches from the Day Reconstruction Method (DRM), which seeks to recover everyday affective sequences through structured episodic recall and specific temporal anchors (Kahneman et al., 2004).

Unlike ecological momentary assessment methodologies (EMA/ESM), which aim to record emotional states in real time, this design sought to approximate the subjective organization of affective work experience through a retrospective sequential reconstruction of a typical workday. This approach allows for the study of intra-daily patterns of well-being and distress in occupational contexts where intensive measurements could interfere with job performance or be operationally difficult to implement (Dockray et al., 2010; Schneider et al., 2020). Therefore, the study did not aim to model complex affective dynamics in real time, but rather to explore subjective temporal trajectories of everyday emotional experience at work.

### 2.2 Participants

The sample comprised 76 healthcare workers who performed direct clinical or patient-care duties in hospital emergency units located in a regional capital in south-central Chile. Participants were recruited from medium and high complexity hospitals using a non-probabilistic convenience sampling procedure based on accessibility and voluntary participation. Eligible participants were required to be actively working in an emergency unit and to perform duties involving direct exposure to the clinical and emotional demands of emergency care.

### 2.3 Instruments

#### 2.3.1 Hourly reconstruction of well-being-distress

A graphic recording sheet, specifically designed to represent the subjective trajectory of well-being and distress over a typical workday, was used. The graphic sheet incorporated a structured hourly axis from T−2 to T14. For the typical daytime shift reconstructed in this study, T0 corresponded approximately to the 08:00 clock-in time and T12 to the 20:00 clock-out time. Accordingly, T−2 and T−1 represented the pre-shift period, T0 through T12 represented the 12-h work shift, and T13 and T14 represented the initial post-shift period. The vertical axis represented an affective continuum from −5 to +5, where negative values indicated varying degrees of subjective distress, positive values indicated subjective well-being, and zero corresponded to affective neutrality.

Participants were asked to place a point for each time slot, indicating how they typically felt during a workday, then connect the points with a continuous line. The instrument’s logic sought to encourage progressive episodic recall through sequential time anchors, reducing nonspecific global responses and allowing for the representation of continuity, deterioration, and affective recovery within the workday. The procedure was developed as an exploratory strategy for subjective temporal reconstruction, not as a momentary, real-time measure of emotional states.

*Burnout* was assessed using the Chilean version of the Burnout Assessment Tool (BAT-4; Hadžibajramović et al., 2024; Maureira-Meneses et al., 2025), which measures the core syndrome through four items rated on a 5-point frequency scale from 1 (never) to 5 (always). In this study, the scale exhibited adequate internal consistency (*ω* = 0.70).

*Emotional dysregulation* was assessed using the Chilean version of the Difficulties in Emotion Regulation Scale (DERS-E; Guzmán-González et al., 2014), a 25-item self-report questionnaire that uses a 5-point Likert scale from 1 (almost never) to 5 (almost always), with higher total scores indicating greater difficulties in emotional regulation. In this study, the scale demonstrated high internal consistency (*ω* = 0.88).

### 2.4 Procedure

Data collection was conducted in group settings within spaces coordinated with the participating organizations. Participants received standardized instructions on how to graphically reconstruct a typical workday using sequential hourly anchors and considering the habitual nature of their affective experience rather than exceptional or isolated episodes. The reconstruction did not include objective or time-specific measures of workload intensity, patient volume, task demands, or exposure to critical events.

Participants were informed that there were no right or wrong answers and that the study aimed to understand subjective patterns of everyday work experience. Subsequently, they completed the supplementary instruments assessing emotional dysregulation and burnout.

Participation was voluntary and confidential. All participants were informed about the study’s general objectives and provided informed consent before participating (CEC-HCHM 202529).

### 2.5 Statistical analysis

Initially, descriptive analyses were conducted to characterize the mean reconstructed trajectory of well-being–distress ratings across the workday. Missing observations were handled through pairwise deletion, such that each correlation was estimated using all participants with valid data for the corresponding pair of variables. Consequently, the effective sample size varied across analyses.

Subsequently, Pearson correlations were calculated between reconstructed time points and between each time point and the complementary psychological variables. Correlations between reconstructed time points were interpreted as indicators of cross-time rank-order consistency; that is, the extent to which participants with relatively higher or lower ratings at one time point tended to maintain their relative position at another time point. These correlations should not be interpreted as direct estimates of within-person affective inertia or moment-to-moment emotional persistence, which would require repeated real-time observations within each participant.

Sex differences at each of the 17 reconstructed time points were examined using independent-samples Student’s *t* tests. The homogeneity of variance assumption was evaluated for each comparison and was not rejected. Cohen’s d was reported as the standardized effect-size measure. Because multiple tests were performed, Benjamini–Hochberg false discovery rate adjustments were calculated separately for four analytic families: correlations involving burnout, correlations involving emotional dysregulation, sex-based comparisons, and cross-time correlations. Both unadjusted *p* values and FDR-adjusted *q* values were examined, with inferential conclusions based on the adjusted results. All analyses were conducted in JASP, version 0.95.4.

Likewise, differentiated associations among burnout, emotional dysregulation, and different times of the workday were explored, with particular attention to the initial, middle, and late phases of the shift. The analyses were interpreted from an exploratory perspective to identify subjective temporal patterns of well-being and distress at work, avoiding causal inferences or direct equivalences with affective processes measured in real time.

## 3 Results

### 3.1 Sample description

The sample consisted of 76 healthcare workers performing direct clinical or patient-care duties in hospital emergency units. The sample included physicians (*n* = 6) and nursing personnel, comprising registered nurses, nursing technicians, and nursing assistants. The gender distribution showed a predominance of women (72.4%), with men accounting for 27.6% of the valid cases. In terms of age, the most frequent group was 30–39 years old (48.7%), followed by 40–49 years old (21.0%). Additionally, 13.6% were aged 50–59, while workers under 30 accounted for 14.4% of the sample.

In terms of job position, professional staff constituted the majority (53.95%), followed by technicians (43.42%), while support staff accounted for only 2.63% of the sample. Most participants reported substantial work experience in emergency services: 63.16% had more than 7 years, whereas only 6.58% had less than 2 years. Collectively, these characteristics describe a predominantly female, professional sample with considerable experience in highly demanding work environments.

### 3.2 Hourly trajectory of well-being–distress

**Figure 1:**
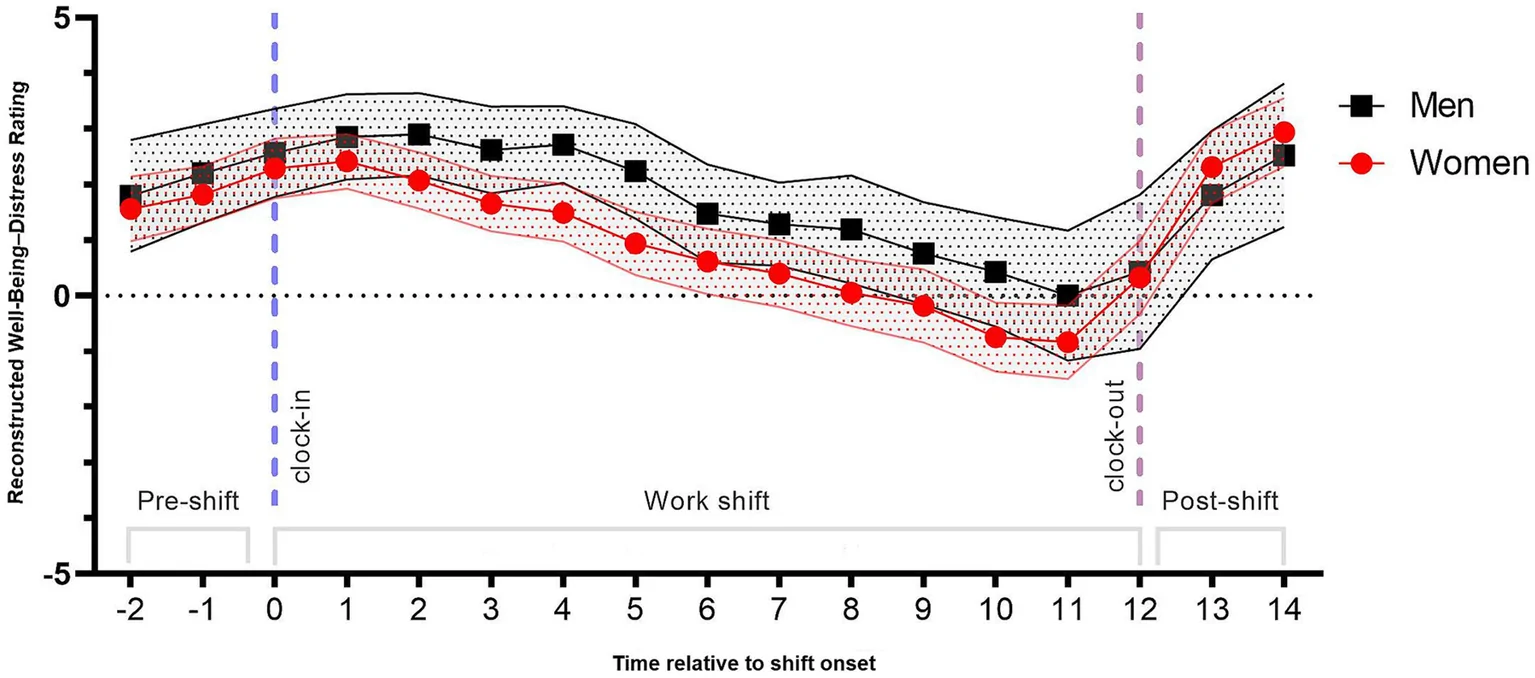
Reconstructed well-being–distress trajectories across a typical daytime shift by sex. Points represent mean retrospectively reconstructed well-being–distress ratings, and shaded areas represent 95% confidence intervals. T0 corresponds to shift onset at 08:00, and T12 corresponds to shift end at 20:00. T−2 and T−1 represent the pre-shift period; T0–T12 represent the work shift; and T13–T14 represent the post-shift period. Sex-specific trajectories are presented descriptively. None of the time-specific sex comparisons remained statistically significant after Benjamini–Hochberg false discovery rate correction.

The average hourly trajectory of well-being–distress exhibited a nonlinear pattern comprising three distinct time segments. As shown in Figure 1, initially, a positive activation phase was observed prior to the formal start of the workday, characterized by a progressive increase in subjective well-being from T−2 (M = 1.63; SD = 2.15) until reaching its maximum levels at T0 (M = 2.37; SD = 1.92) and T1 (M = 2.54; SD = 1.78). This pattern suggests a relatively favorable anticipatory period preceding the start of the work shift.

Subsequently, the curve changed direction between T1 and T2, followed by a progressive decrease in reconstructed affective valence throughout much of the shift. Mean ratings declined from T2 (M = 2.30, SD = 1.84) to their lowest value at T11 (M = −0.61, SD = 2.50), indicating a shift from positive well-being ratings toward greater subjectively reconstructed distress. This decline was accompanied by increased between-participant variability, with standard deviations approaching or exceeding two scale points at several moments of the shift.

Finally, from T12 (M = 0.36, SD = 2.60) onward, the mean trajectory changed direction and returned to positive values at T13 (M = 2.17, SD = 2.37) and T14 (M = 2.82, SD = 2.36), exceeding the mean ratings observed during the pre-shift period. This descriptive increase indicates a more favorable reconstructed affective evaluation during the post-shift period but should not be interpreted as direct evidence of psychological recovery from emotional exhaustion. Descriptive statistics and sex-based comparisons at each reconstructed time point are presented in Table 1.

**Table 1:** Sex-based comparisons and correlations of reconstructed well-being–distress ratings across the shift.

Time point	Men		Women		*t*	df	*p*	*q*	*d*	ED		BO	
	M	SD	M	SD						*r*	*q*	*r*	*q*
T−2	1.80	2.14	1.56	2.17	0.42	73	0.676	0.719	0.11	0.10	0.744	−0.02	0.922
T−1	2.20	1.88	1.82	1.89	0.78	73	0.440	0.576	0.20	0.01	0.983	−0.01	0.922
T0 (shift onset)	2.57	1.75	2.29	1.99	0.57	74	0.572	0.648	0.15	−0.13	0.591	−0.11	0.465
T1	2.86	1.68	2.42	1.82	0.96	74	0.341	0.527	0.25	−0.17	0.480	−0.15	0.337
T2	2.90	1.64	2.07	1.87	1.79	74	0.078	0.221	0.46	−0.15	0.514	−0.26	0.116
T3	2.62	1.72	1.65	1.86	2.06	74	0.042	0.162	0.53	−0.20	0.455	−0.19	0.276
T4	2.71	1.52	1.49	1.90	2.64	74	0.010	0.138	0.68	−0.20	0.455	−0.25	0.116
T5	2.24	1.87	0.95	2.11	2.46	74	0.016	0.138	0.63	−0.24	0.455	−0.15	0.337
T6	1.48	1.94	0.62	2.19	1.57	74	0.120	0.226	0.40	−0.02	0.983	−0.03	0.891
T7	1.29	1.65	0.40	2.22	1.66	74	0.102	0.226	0.42	0.02	0.983	−0.13	0.433
T8	1.19	2.14	0.05	2.22	2.01	74	0.048	0.162	0.52	−0.13	0.591	−0.18	0.276
T9	0.76	2.02	−0.18	2.44	1.57	74	0.120	0.226	0.40	−0.17	0.480	−0.32	0.074
T10	0.43	2.16	−0.75	2.29	2.03	74	0.046	0.162	0.52	−0.01	0.983	−0.15	0.337
T11	0.00	2.57	−0.84	2.46	1.31	74	0.195	0.331	0.34	−0.08	0.796	−0.26	0.116
T12 (shift end)	0.43	3.04	0.33	2.43	0.15	74	0.880	0.880	0.04	−0.06	0.908	−0.19	0.276
T13	1.81	2.54	2.31	2.31	−0.82	70	0.416	0.576	−0.21	−0.03	0.983	−0.10	0.539
T14	2.52	2.84	2.94	2.15	−0.67	69	0.502	0.610	−0.18	0.00	0.983	−0.06	0.776

ED, emotional dysregulation; BO, burnout; *d* = Cohen’s standardized mean difference. Sex-based comparisons were conducted using independent-samples Student’s *t* tests. The homogeneity-of-variance assumption was not rejected at any time point. The *p* values are unadjusted; *q* values were adjusted using the Benjamini–Hochberg false discovery rate procedure. Adjustments were calculated separately for the 17 sex comparisons, 17 ED correlations, and 17 BO correlations. No sex comparison or correlation with ED or BO remained statistically significant at *q* < 0.05.

### 3.3 Cross-time rank-order consistency

Cross-time correlations were generally strongest between adjacent reconstructed time points and decreased as the temporal distance between points increased (Table 2). All 16 adjacent-time correlations remained statistically significant after FDR correction. The largest adjacent associations were observed between T−2 and T−1 (*r* = 0.86, *p* < 0.001), T1 and T2 (*r* = 0.84, *p* < 0.001), T2 and T3 (*r* = 0.84, *p* < 0.001), T8 and T9 (*r* = 0.86, *p* < 0.001), T10 and T11 (*r* = 0.78, *p* < 0.001), and T13 and T14 (*r* = 0.78, *p* < 0.001).

**Table 2:** Cross-time correlations of reconstructed well-being–distress ratings by temporal lag.

Time	Lag 1	Lag 2	Lag 3	Lag 4	Lag 5	Lag 6
T−2	0.86^***^	0.55^***^	0.43^***^	0.32^*^	0.17	0.05
T−1	0.72^***^	0.54^***^	0.40^***^	0.31^*^	0.17	0.09
T0	0.72^***^	0.62^***^	0.55^***^	0.40^***^	0.12	0.00
T1	0.84^***^	0.70^***^	0.51^***^	0.27^*^	0.08	0.10
T2	0.84^***^	0.71^***^	0.40^***^	0.24	0.26^*^	0.12
T3	0.82^***^	0.52^***^	0.37^**^	0.28^*^	0.20	0.19
T4	0.60^***^	0.44^***^	0.35^**^	0.28^*^	0.23	0.12
T5	0.76^***^	0.50^***^	0.41^***^	0.38^**^	0.19	0.01
T6	0.69^***^	0.50^***^	0.42^***^	0.30^*^	0.05	−0.03
T7	0.76^***^	0.64^***^	0.46^***^	0.27^*^	0.06	−0.15
T8	0.86^***^	0.64^***^	0.42^***^	0.13	−0.09	−0.08
T9	0.80^***^	0.62^***^	0.25^*^	−0.07	−0.10	—
T10	0.78^***^	0.37^**^	0.10	0.00	—	—
T11	0.62^***^	0.24	0.10	—	—	—
T12	0.52^***^	0.33^**^	—	—	—	—
T13	0.78^***^	—	—	—	—	—

Values are Pearson correlations between reconstructed well-being–distress ratings at different time points. Lag represents the temporal distance, in hours, between paired reconstructed ratings. Benjamini–Hochberg false discovery rate correction was applied across the 81 cross-time correlations. Fifty-two correlations remained statistically significant at *q* < 0.05, including all 16 correlations between adjacent time points. Effective sample sizes ranged from 71 to 76 because of missing observations. Strong adjacent-time correlations indicate cross-time rank-order consistency in participants’ retrospective ratings and should not be interpreted as direct estimates of within-person affective inertia or real-time temporal continuity. Dashes indicate correlations that could not be estimated because the corresponding subsequent time points were unavailable. ^*^q < 0.05, ^**^q < 0.01, ^***^q < 0.001.

Across all temporal distances, 52 of the 81 cross-time correlations remained statistically significant after FDR correction. The reduction in correlation magnitude at longer temporal lags indicates that participants’ relative ordering was more consistent between nearby reconstructed moments than between temporally distant moments.

These findings provide exploratory support for H1. Within the retrospective reconstruction task, participants’ relative positions in well-being–distress ratings tended to be more consistent across nearby than across temporally distant moments.

### 3.4 Associations between burnout, emotional dysregulation and work schedule

At the unadjusted level, exploratory correlations between burnout and reconstructed well-being–distress ratings were observed at T2, T4, T9, and T11. The largest association occurred at T9 (*r* = −0.32, *p* = 0.004) although it did not remain statistically significant after FDR adjustment (*q* = 0.074). The remaining nominal associations were small and likewise did not survive correction. Emotional dysregulation showed one nominal association at T5 (*r* = −0.24, *p* = 0.033, *q* = 0.455). Thus, the corrected analyses did not provide statistically robust evidence of time-specific associations involving burnout or emotional dysregulation.

Accordingly, neither H2 nor H3 was supported after correction for multiple comparisons. The unadjusted correlations are retained as descriptive, hypothesis-generating results and should not be interpreted as evidence of reliable time-specific associations.

At the unadjusted level, the sex comparisons yielded nominal *p* values below 0.05 at T3, T4, T5, T8, and T10, with moderate effect-size estimates. However, none of the 17 time-specific comparisons remained statistically significant after FDR correction; the smallest adjusted value was *q* = 0.138 at T4 and T5. Accordingly, the analyses did not provide statistically robust evidence of sex differences at specific reconstructed time points.

## 4 Discussion

The reconstructed trajectory is consistent with affective-dynamics research showing that affective experience can vary over time rather than remaining static (Houben et al., 2015; Koval et al., 2013). In the present study, participants retrospectively represented a typical emergency shift as a sequence of relatively favorable pre-shift ratings, a gradual decline across much of the work period, and a subsequent increase during the post-shift period. This descriptive pattern highlights the value of situating subjective affective experience within the temporal organization of work rather than examining it exclusively through global person-level indicators.

The cross-time correlations provide exploratory support for H1. All 16 correlations between adjacent reconstructed time points remained statistically significant after FDR correction, and the magnitude of the associations generally decreased as the temporal distance between points increased. Consistent with research emphasizing temporal dependencies and the importance of the measurement timescale in affective dynamics (Nelson et al., 2020; Schneider et al., 2020), this pattern suggests that participants’ relative positions in reconstructed well-being–distress ratings were more consistent across nearby than across distant moments. In this sense, the findings indicate short-range rank-order consistency within the reconstructed profiles, while the mean trajectory continued to change across the shift. This pattern provides an empirical basis for future intensive longitudinal studies examining whether comparable temporal organization is observed in within-person affective persistence measured in real time.

Regarding the person-level psychological variables, the unadjusted analyses showed negative correlations between burnout and reconstructed well-being–distress ratings at several time points. However, none of these associations remained statistically significant after FDR correction, and H2 was therefore not supported. Similarly, the nominal association involving emotional dysregulation did not survive correction, providing no corrected statistical support for H3. These results do not contradict evidence that burnout is associated with sustained occupational overload and less favorable well-being outcomes (Salvagioni et al., 2017; Edú-Valsania et al., 2022); rather, they indicate that the present exploratory data do not provide sufficiently robust evidence to identify specific periods of the shift in which burnout or emotional dysregulation is more strongly associated with reconstructed affective experience. The nominal patterns may inform hypotheses for future studies with larger samples and intensive longitudinal measurements.

The sex-based analyses did not provide statistically significant evidence of differences at specific reconstructed time points after FDR correction. Although moderate unadjusted effect-size estimates were observed at several moments, these patterns were not sufficiently robust to support conclusions about sex-specific temporal differences. Moreover, their apparent temporal overlap with the nominal burnout and emotional dysregulation correlations should not be interpreted substantively because none of these families of associations remained significant after correction. Sex-related variation in reconstructed intra-shift affective experience therefore remains an open question for studies with larger and more balanced samples.

From a workers’ mental health perspective, understanding how affective experience is reconstructed across the workday may provide a useful temporal framework for designing brief organizational micro-interventions. This possibility is particularly relevant in emergency and hospital settings, where operational demands constrain the duration and timing of worker-support initiatives. The less favorable mean ratings observed during several segments of the shift suggest that brief recovery opportunities, timely supervisory support, team check-ins, or temporary task adjustments could be distributed at strategically selected moments of the workday. However, because workload, task exposure, and critical events were not measured, the present findings should be regarded as identifying candidate periods for future evaluation rather than establishing definitive temporal vulnerability windows or prescribing optimal intervention times.

Several limitations should be considered. First, the study relied on a single retrospective reconstruction of a typical work shift rather than intensive real-time assessments. Consequently, the reported trajectories may be affected by recall bias, social desirability, and participants’ generalized representations of their work experience. The cross-sectional design also prevents causal inference, the examination of within-person change in real time, or the identification of mechanisms underlying the reconstructed patterns.

Second, the graphic reconstruction procedure was developed as an exploratory structured recall tool and has not been formally validated as a psychometric measure. Consequently, the observed trajectories should be interpreted as participants’ subjective retrospective representations of a typical work shift rather than as direct or psychometrically validated measurements of momentary affect. Furthermore, the study did not assess time-specific workload, patient volume, task demands, exposure to critical events, sleep, chronotype, light exposure, or physiological circadian markers (English and Carstensen, 2014; Tucker et al., 2012; Walker II et al., 2020). Therefore, the observed pattern cannot determine whether changes in reconstructed affective experience reflect the temporal organization of work demands, time-of-day influences, circadian processes, or a combination of these factors.

Third, no *a priori* sample-size calculation was conducted. The modest, non-probabilistic sample was recruited from emergency services in a specific national and occupational context, limiting statistical precision and generalizability. In addition, the number of time-specific correlations and group comparisons increased the risk of Type I error. After applying false discovery rate corrections, none of the associations involving burnout, emotional dysregulation, or sex remained statistically significant, although all correlations between adjacent reconstructed time points remained statistically significant after FDR correction. Accordingly, isolated associations based on unadjusted *p*-values should be interpreted as nominal and hypothesis-generating rather than as evidence of distinct temporal effects.

Finally, the sex distribution reflects the documented feminization of the healthcare workforce (Li et al., 2024), and an artificial 1:1 balance was therefore not pursued. Nevertheless, the smaller number of men limited the precision of the sex-based comparisons (Shannon et al., 2019). These comparisons should be regarded as exploratory descriptive patterns and require replication in larger and more balanced samples.

Future research should examine these preliminary patterns using larger and more diverse occupational samples, validated temporal affect measures, and intensive longitudinal designs such as EMA or ESM. These studies should integrate clock-time registration, workload and patient-volume indicators, task and critical-event logs, and relevant individual factors such as sleep and chronotype. This would allow researchers to distinguish changes associated with the temporal organization of work from time-of-day or circadian influences. Future studies should also test whether the candidate periods suggested by the reconstructed trajectory can inform the timing of brief organizational micro-interventions, including recovery opportunities, supervisory support, team check-ins, or temporary task adjustments during emergency shifts.

## Data Availability

The datasets presented in this article are not readily available because they contain information derived from human participants and are subject to ethical and confidentiality considerations. Data may be made available by the authors upon reasonable request, provided that the request is scientifically justified and consistent with applicable ethical requirements. Requests to access the datasets should be directed to Sebastián Maureira-Meneses, smaureira@ubiobio.cl.

## References

[ref1] Alanazy A. Alruwaili A. (2023). The global prevalence and associated factors of burnout among emergency department healthcare workers and the impact of the COVID-19 pandemic: a systematic review and meta-analysis. Healthcare (Basel) 11:2220. doi: 10.3390/healthcare11152220, 37570460 PMC10418606

[ref2] Anusic I. Lucas R. (2016). The validity of the day reconstruction method in the German socio-economic panel study. Soc. Indic. Res. 130, 213–232. doi: 10.1007/s11205-015-1172-6, 30532344 PMC6286067

[ref3] Bakker A. Demerouti E. Oerlemans W. Sonnentag S. (2012). Workaholism and daily recovery: a day-reconstruction study of leisure activities. J. Organ. Behav. 34, 87–107. doi: 10.1002/job.1796, 42587375

[ref4] Boemo T. Nieto I. Vázquez C. Sánchez-López Á. (2022). Relations between emotion regulation strategies and affect in daily life: a systematic review and Meta-analysis of studies using ecological momentary assessments. Neurosci. Biobehav. Rev. 139:104747. doi: 10.1016/j.neubiorev.2022.104747, 35716875

[ref5] Brose A. Schmiedek F. Gerstorf D. Voelkle M. (2020). The measurement of within-person affect variation. Emotion 20, 677–699. doi: 10.1037/emo0000583, 31008618

[ref6] Cohen C. Pignata S. Bezak E. Tie M. Childs J. (2023). Workplace interventions to improve well-being and reduce burnout for nurses, physicians, and allied healthcare professionals: a systematic review. BMJ Open 13:e071203. doi: 10.1136/bmjopen-2022-071203, 37385740 PMC10314589

[ref7] Diao D. Chen X. Zhong L. Zhang H. Zhang J. (2024). Sex differences in burnout and work-family conflict among Chinese emergency nurses: a cross-sectional study. Front. Public Health 12:1492662. doi: 10.3389/fpubh.2024.1492662, 39712298 PMC11659251

[ref8] Diener E. Tay L. (2014). Review of the day reconstruction method (DRM). Soc. Indic. Res. 116, 255–267. doi: 10.1007/s11205-013-0279-x

[ref9] Diener E. Thapa S. Tay L. (2020). Positive emotions at work. Annu. Rev. Organ. Psychol. Organ. Behav. 7, 451–477. doi: 10.1146/annurev-orgpsych-012119-044908

[ref10] Dockray S. Grant N. Stone A. Kahneman D. Wardle J. Steptoe A. (2010). A comparison of affect ratings obtained with ecological momentary assessment and the day reconstruction method. Soc. Indic. Res. 99, 269–283. doi: 10.1007/s11205-010-9578-7, 21113328 PMC2990978

[ref11] Doran K. Witmer S. Yoon K. Fischer E. Ebangwese A. Sharma S. . (2023). Gauging the stress of long-term care nursing assistants using ecological momentary assessment, wearable sensors, and end-of-day reconstruction. Int. J. Older People Nursing 19:e12592. doi: 10.1111/opn.12592, 38098142

[ref12] Edú-Valsania S. Laguía A. Moriano J. (2022). Burnout: a review of theory and measurement. Int. J. Environ. Res. Public Health 19:1780. doi: 10.3390/ijerph19031780, 35162802 PMC8834764

[ref13] English T. Carstensen L. L. (2014). Emotional experience in the mornings and the evenings: consideration of age differences in specific emotions by time of day. Front. Psychol. 5:Article 185. doi: 10.3389/fpsyg.2014.00185, 24639663 PMC3944144

[ref14] Guzmán-González M. Trabucco C. Urzúa A. Garrido L. Leiva J. (2014). Validez y confiabilidad de la versión adaptada al español de la escala de dificultades de regulación emocional (DERS-E) en población chilena. Ter. Psicol. 32, 19–29. doi: 10.4067/S0718-48082014000100002

[ref15] Hadžibajramović E. Schaufeli W. B. De Witte H. (2024). The ultra-short version of the burnout assessment tool (BAT4)—development, validation, and measurement invariance across countries, age and gender. PLoS One 19:e0297843. doi: 10.1371/journal.pone.0297843, 38394265 PMC10889892

[ref16] Houben M. Van Den Noortgate W. Kuppens P. (2015). The relation between short-term emotion dynamics and psychological well-being: a meta-analysis. Psychol. Bull. 141, 901–930. doi: 10.1037/a0038822, 25822133

[ref17] Izdebski Z. Kozakiewicz A. Białorudzki M. Dec-Pietrowska J. Mazur J. (2023). Occupational burnout in healthcare workers, stress and other symptoms of work overload during the COVID-19 pandemic in Poland. Int. J. Environ. Res. Public Health 20:2428. doi: 10.3390/ijerph20032428, 36767797 PMC9916221

[ref18] Kahneman D. Krueger A. Schkade D. Schwarz N. Stone A. (2004). A survey method for characterizing daily life experience: the day reconstruction method. Science 306, 1776–1780. doi: 10.1126/science.1103572, 15576620

[ref19] Kim J. Kikuchi H. Yamamoto Y. (2013). Systematic comparison between ecological momentary assessment and day reconstruction method for fatigue and mood states in healthy adults. Br. J. Health Psychol. 18, 155–167. doi: 10.1111/bjhp.12000, 23017062

[ref20] Koval P. M. Meers K. Kuppens P. (2013). Affect dynamics in relation to depressive symptoms: variable, unstable, or inert? Emotion 13, 1132–1141. doi: 10.1037/a0033579, 23914765

[ref21] Li M. Raven J. Liu X. (2024). Feminization of the health workforce in China: exploring gendered composition from 2002 to 2020. Hum. Resour. Health 22:15. doi: 10.1186/s12960-024-00898-w, 38373975 PMC10877893

[ref22] Lucas R. E. Wallsworth C. Anusic I. Donnellan M. B. (2021). A direct comparison of the day reconstruction method (DRM) and the experience sampling method (ESM). J. Pers. Soc. Psychol. 120, 816–835. doi: 10.1037/pspp0000289, 32202810 PMC7508776

[ref23] Maureira-Meneses S. Mendoza-Llanos R. Campos-Gutiérrez M. Valladares Reyes S. (2025). Propiedades psicométricas y estructura factorial del Burnout Assessment Tool-12 en trabajadores del centro-sur de Chile. Psykhe, 35, 1–1. doi: 10.7764/psykhe.2025.SCP102712

[ref24] Nagle E. Griškēviča I. Rajevska O. Ivanovs A. Mihailova S. Skrūzkalne I. (2024). Factors affecting healthcare workers’ burnout and their conceptual models: scoping review. BMC Psychol. 12:637. doi: 10.1186/s40359-024-02130-9, 39511697 PMC11545506

[ref25] Nelson J. Klumparendt A. Doebler P. Ehring T. (2020). Everyday emotional dynamics in major depression. Emotion 20, 179–191. doi: 10.1037/emo0000541, 30589297

[ref26] Purvanova R. Muros J. (2010). Gender differences in burnout: a meta-analysis. J. Vocat. Behav. 77, 168–185. doi: 10.1016/j.jvb.2010.04.006

[ref27] Salvagioni D. Melanda F. Mesas A. González A. Gabani F. Andrade S. (2017). Physical, psychological, and occupational consequences of job burnout: a systematic review of prospective studies. PLoS One 12:e0185781. doi: 10.1371/journal.pone.0185781, 28977041 PMC5627926

[ref28] Schneider S. Junghaenel D. Gutsche T. Mak H. Stone A. (2020). Comparability of emotion dynamics derived from ecological momentary assessments, daily diaries, and the day reconstruction method: observational study. J. Med. Internet Res. 22:e19201. doi: 10.2196/19201, 32969835 PMC7545330

[ref29] Shannon G. Minckas N. Tan D. Haghparast-Bidgoli H. Batura N. Mannell J. (2019). Feminisation of the health workforce and wage conditions of health professions: an exploratory analysis. Hum. Resour. Health 17:72. doi: 10.1186/s12960-019-0406-0, 31623619 PMC6796343

[ref30] Tucker A. M. Feuerstein R. Mende-Siedlecki P. Ochsner K. N. Stern Y. (2012). Double dissociation: circadian off-peak times increase emotional reactivity; aging impairs emotion regulation via reappraisal. Emotion 12, 869–874. doi: 10.1037/a0028207, 22642354 PMC3758763

[ref31] Walker W. H. II Walton J. C. DeVries A. C. Nelson R. J. (2020). Circadian rhythm disruption and mental health. Transl. Psychiatry 10:Article 28. doi: 10.1038/s41398-020-0694-0, 32066704 PMC7026420

